# Impact of surgical margin status on the outcome of bladder cancer treated by radical cystectomy: a meta-analysis

**DOI:** 10.18632/oncotarget.12907

**Published:** 2016-10-25

**Authors:** Xuwei Hong, Tieqiu Li, Fengsheng Ling, Dashan Yang, Lina Hou, Fei Li, Wanlong Tan

**Affiliations:** ^1^ Department of Urology, Nanfang Hospital, Southern Medical University, Guangzhou, Guangdong, P. R. China; ^2^ Department of Urology, The Peoples Hospital of Hunan Province, First Affiliated Hospital of Hunan Normal University, Changsha, P. R. China; ^3^ Department of Healthy Management, Nanfang Hospital, Southern Medical University, Guangzhou, Guangdong, P. R. China

**Keywords:** bladder cancer, surgical margin status, radical cystectomy, outcome, meta-analysis

## Abstract

Data regarding the association between surgical margin status and the outcome of bladder cancer treated by radical cystectomy (RC) are conflicting. Therefore, the present meta-analysis was performed to assess the associations between the outcomes of bladder cancer, in terms of recurrence-free survival (RFS), cancer-specific survival (CSS) and overall survival (OS), and the presence of positive surgical margins versus negative surgical margins following treatment with RC. Research articles published prior to April 2016 were identified from Pubmed, Embase and the Cochrane Library databases. A total of 36 articles were included, with a sample size of 38,384 bladder cancer patients. Of these, 4,354 patients were reported to have positive surgical margins. Significant associations were detected between positive surgical margins following RC and unfavorable RFS [summary relative risk estimate (SRRE), 1.63; 95% confidence interval (CI), 1.46-1.83; *P* = 0.105], CSS (SRRE, 1.82; 95% CI, 1.63-2.04; *P* = 0.001) and OS (SRRE, 1.68; 95% CI, 1.58-1.80; *P* = 0.805), by fixed or random effects models. The findings were consistent independently of age, sample size, publication year, follow-up duration, study type and geographical region. In summary, the present findings demonstrate that the presence of positive surgical margins is associated with poor survival outcomes in bladder cancer following RC, indicating that avoidance of positive surgical margins during surgery is helpful to improve the prognosis of patients with bladder cancer.

## INTRODUCTION

Bladder cancer is the ninth most commonly diagnosed cancer worldwide and ranks second in terms of incidence among genitourinary malignancies. The American Cancer Society estimated that in the United States there were 76,960 new cases and 16,390 mortalities from bladder cancer in 2016 [1]. A trend of increasing incidence and mortality rates has been observed in the past three decades. Radical cystectomy (RC) is the standard treatment for muscle-invasive bladder cancer and non-muscle-invasive bladder cancers with high-risk features [2]. Although minimally invasive techniques have increased in application, with the goal of minimizing patient mortality, the mortality associated with bladder cancer following RC has not changed substantially in the last 30 years [3]. Research regarding the effects of risk factors on the survival outcomes of bladder cancer following treatment with RC remains important. Currently, the stage and grade of tumors are used as the major prognostic factors for these patients [3]. However, there is growing interest in identifying additional prognostic indicators to aid medical professionals in improving prognostic evaluation.

Surgical margin status is determined by the presence or absence of tumor tissue in the areas of soft tissue around the surgical specimen. Although the potential associations between surgical margin status and survival outcome have received much attention in the past few years, studies have yielded inconsistent results. Several large retrospective studies have demonstrated that positive surgical margins are an independent predictor of recurrence and eventual mortality from bladder cancer [4–6]. On the contrary, other studies demonstrated that positive surgical margins were not independently associated with the risk of local recurrence or the disease-free survival of bladder cancer patients after RC, indicating that surgical margin status may not be a significant factor in determining the eventual prognosis compared with other widely accepted prognostic indicators [7–9]. To date, no quantitative assessment concerning the association of surgical margin status with outcomes in bladder cancer following treatment with RC has been conducted.

In the present study, a meta-analysis was conducted to summarize the relationship between surgical margin status and bladder cancer outcome after RC based on all published epidemiological studies. The purpose of this meta-analysis was to clarify the association between surgical margin status and survival outcomes of RC, and explore potential sources of heterogeneity across different studies.

## RESULTS

### Search results

**Figure 1:**
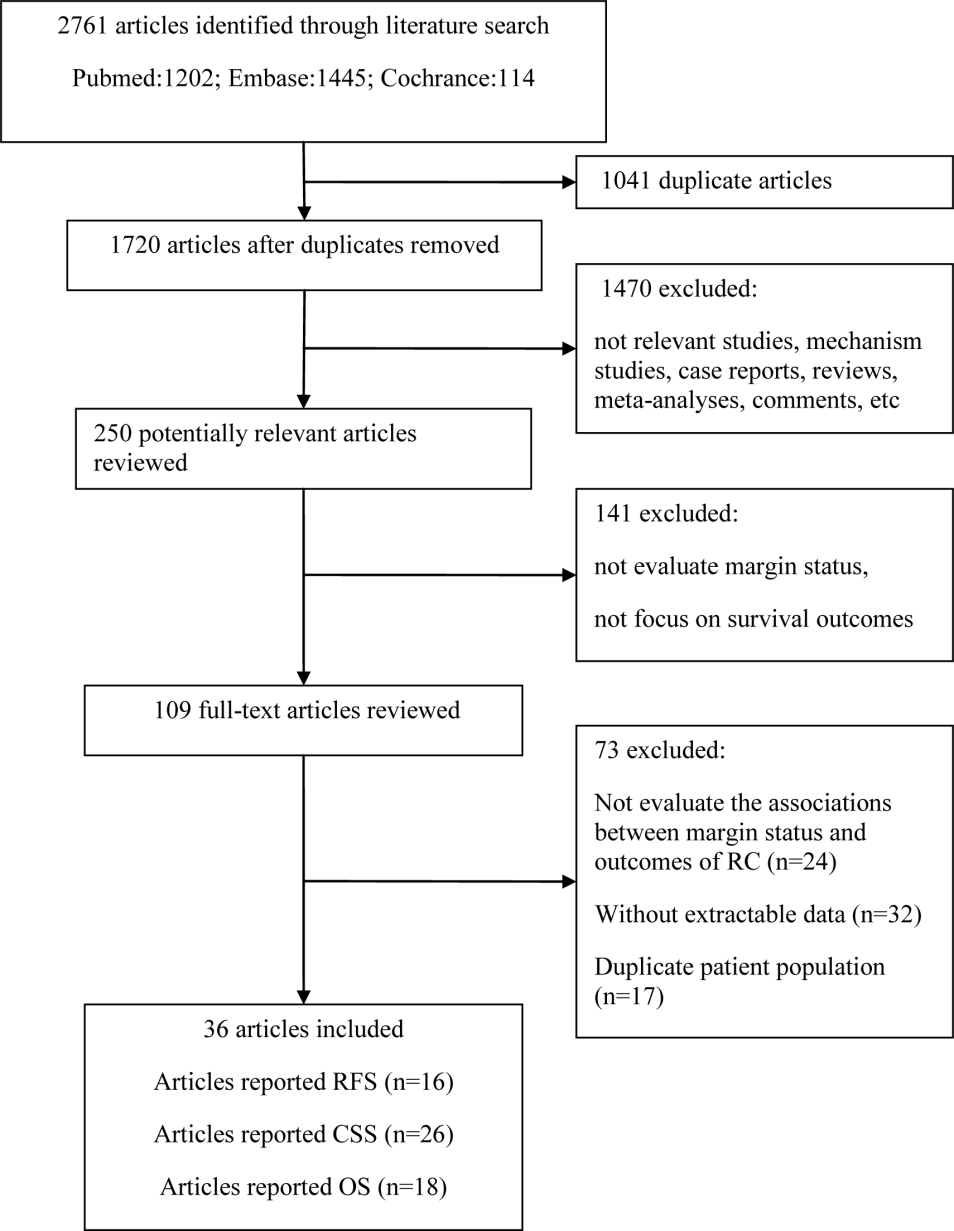
Flow chart illustrating the study selection

Figure 1 illustrates the process of the literature search. Briefly, a total of 2,761 articles were identified using our search strategy, of which 250 were considered potentially relevant articles after excluding duplicate articles and screening the titles and abstracts. A further 141 articles were excluded because that they did not evaluate margin status, or did not focus on survival outcomes. After assessing the remaining 109 articles by full-text review, 73 articles were excluded; of these, 24 articles did not investigate the associations between surgical margin status and survival outcomes of bladder cancer in patients who had undergone RC, 32 were excluded because they did not report relative risk estimates and the corresponding 95% CI or did not provide the sufficient data to calculate them, and 17 articles were excluded as the participants overlapped with other studies. Finally, a total of 36 articles were included in the meta-analysis. The outcomes used were recurrence-free survival (RFS) in 16 studies, cancer-specific survival (CSS) in 26 studies and overall survival (OS) in 18 studies.

### Characteristics of the included studies

The main characteristics of the included studies are shown in Table 1. All studies were published between 2004 and 2015, with the mean duration of follow-up varying from 20 to 148 months. Of the 36 studies, 14 were conducted in North America [4, 8–20], 8 in Europe [21–28], 6 in Asia [7, 29–33] and 8 at international multicenters [34–41]. The meta-analysis was based on a total sample size of 38,384 patients, of which 4,354 patients were reported to have positive surgical margins. Regarding tumor stage, 19,377 patients presented with organ-confined disease and 18,669 with non-organ confined disease. The majority of the included studies were limited to urothelial bladder carcinoma, while 12 studies involved other tumor types, including squamous cell carcinoma, adenocarcinoma and small cell carcinoma. In addition, there were 5 studies [11, 14, 15, 20, 29] in which the histopathological types were not described.

**Table 1:** Characteristics of studies included in meta-analysis of surgical margin status and bladder cancer outcomes

Study	Country/Type	Period	Mean follow -up(months)	Mean age	Sample-size	Positive SM(%)	Positive LN(%)	T stage(%)
Soave 2015 [21]	Germany; single-center study	1996-2011	45.0	67.0	517	12.0	26.7	≤T2:55.5; ≥T3:44.5
Satkunasivam 2015 [10]	USA; single-center study	1971-2009	148.8	66.2	2047	0.0	NA	≤T2:63.3; ≥T3:36.7
Reder 2015 [8]	USA; single-center study	2000-2012	20.0	67.9	364	10.7	20.0	≤T2:54.1; ≥T3:44.5
Raza 2015 [34]	International multicenter study	2003-2015	67.0	69.0	702	8.0	21.0	≤T2:62.0; ≥T3:38.0
Kanatani 2015 [29]	Japan; single-center study	1990-2012	29.0	64.0	61	11.5	50.8	≤T2:13.1; ≥T3:86.9
Gakis 2015 [35]	International multicenter study	1994-2011	64.0	54.0	297	2.4	20.2	≤T2:40.4; ≥T3:59.6
Booth 2015 [11]	Canada; single-center study	1994-2008	NA	72.0	2802	13.0	29.0	≤T2:29.0; ≥T3:71.0
Aziz 2015 [36]	International multicenter study	1989-2011	40.0	68.0	856	24.8	53.6	≤T2:0.0; ≥T3:100.0
Albisinni 2015 [22]	International multicenter study	2000-2013	50.0	68.0	503	5.8	23.1	≤T2:57.9; ≥T3:42.1
Yuh 2014 [12]	USA; single-center study	2004-2012	52.0	70.0	162	4.3	21.6	≤T2:66.7; ≥T3:33.3
Suer 2014 [30]	Turkey; single-center study	1990-2012	37.7	66.5	290	7.6	14.5	≤T2:54.8; ≥T3:45.2
Sejima 2014 [31]	Japan; single-center study	2003-2011	24.8	71.1	249	4.4	15.7	≤T2:56.6; ≥T3:43.4
Ploussard 2014 [37]	International multicenter study	1979-2012	32.2	68.0	8141	23.7	23.7	≤T2:56.8; ≥T3:43.2
Nieuwenhuijzen 2014 [23]	Netherlands; single-center study	1990-2006	64.0	62.3	343	10.0	34.0	≤T2:52.0; ≥T3:48.0
May 2014 [24]	International multicenter study	1989-2008	36.0	67.0	385	22.3	51.4	≤T2:0.0; ≥T3:100.0
Lin 2014 [12]	USA; single-center study	1990-2010	66.0	68.0	196	0.0	NA	≤T2:100.0; ≥T3:0.0
Kluth 2014 [38]	International multicenter study	1998-2010	36.1	67.0	2895	5.5	26.9	≤T2:53.9; ≥T3:46.1
Klatte 2014 [39]	International multicenter study	1979-2012	41.0	67.5	7906	5.3	23.8	≤T2:58.1; ≥T3:41.9
Bruins 2014 [25]	Netherlands; single-center study	1998-2011	75.6	65.0	245	2.9	NA	≤T2:64.9; ≥T3:35.1
Bachir 2014 [14]	Canada; multicenter study	1998-2008	39.0	65.6	847	10.6	22.4	≤T2:49.8; ≥T3:50.2
Lotan 2013 [9]	USA; single-center study	2007-2012	20.0	70.0	216	7.0	25.0	≤T2:60.0; ≥T3:40.0
Fritsche 2013 [26]	Germany; multicenter study	2006-2010	20.0	69.0	158	26.6	100.0	≤T2:19.6; ≥T3:80.4
Todenhofer 2012 [27]	Germany; single-center study	1999-2010	30.0	67.8	258	10.1	27.1	≤T2:50.4; ≥T3:49.6
Mitra 2012 [15]	USA; single-center study	1971-2005	31.2	62.3	447	7.8	48.8	≤T2:26.6; ≥T3:24.6
Gondo 2012 [32]	Japan; single-center study	2000-2009	26.8	68.0	194	10.3	10.8	≤T2:55.7; ≥T3:44.3
Yafi 2011 [16]	Canada; multicenter study	1998-2008	35.0	68.0	2287	8.6	25.9	≤T2:48.1; ≥T3:51.9
Sonpavde 2011 [40]	International multicenter study	1971-2008	39.4	68.5	578	4.0	0.0	≤T2:0.0; ≥T3:100.0
Hofner 2011 [28]	Germany; single-center study	1990-2009	104.4	64.0	328	17.0	36.0	≤T2:49.0; ≥T3:51.0
Tilki 2010 [41]	International multicenter study	1979-2008	55.0	68.9	583	24.9	53.5	≤T2:0.0; ≥T3:100.0
Kim 2010 [33]	Korea; single-center study	1986-2005	66.3	60.8	406	3.9	12.1	≤T2:67.2; ≥T3:32.8
Fairey 2009 [17]	Canada; single-center study	1994-2007	31.0	66.0	523	12.0	23.0	≤T2:49.0; ≥T3:51.0
Chapman 2009 [18]	USA; single-center study	1996-2006	34.3	66.4	308	12.7	27.3	≤T2:49.0; ≥T3:51.0
Canter 2009 [19]	USA; single-center study	1988-2006	46.4	65.5	344	11.6	NA	≤T2:89.0; ≥T3:11.0
Dotan 2007 [4]	USA; single-center study	1985-2005	NA	65.9	1589	4.2	24.0	≤T2:54.0; ≥T3:46.0
Lee 2006 [7]	Korea; single-center study	1995-2002	37.1	61.0	115	4.3	0.0	NA
Herr 2004 [20]	USA; multicenter study	1987-1998	106.8	64.6	242	10.0	20.5	≤T2:69.0; ≥T3:31.0

Abbreviations: SM, surgical margin; LN, lymph node; NA, data not applicable

### Meta-analysis

**Figure 2:**
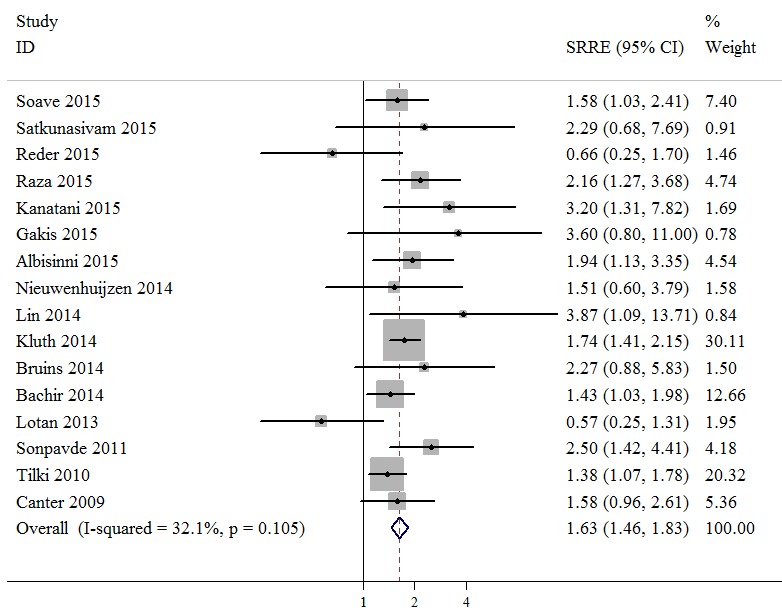
Meta-analysis of studies that examined the association between positive surgical margin and recurrence-free survival (RFS) following radical cystectomy (RC)

**Figure 3:**
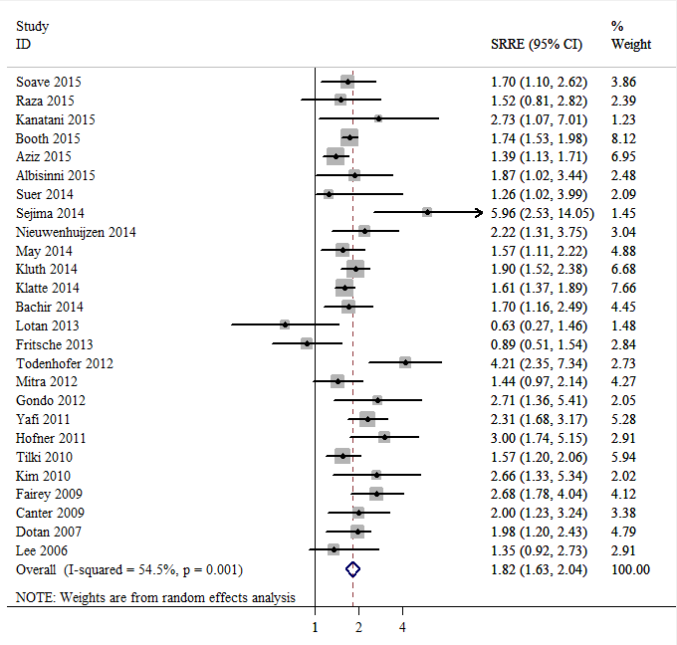
Meta-analysis of studies that examined the association between positive surgical margin and cancer-specific survival (CSS) following radical cystectomy (RC)

**Figure 4:**
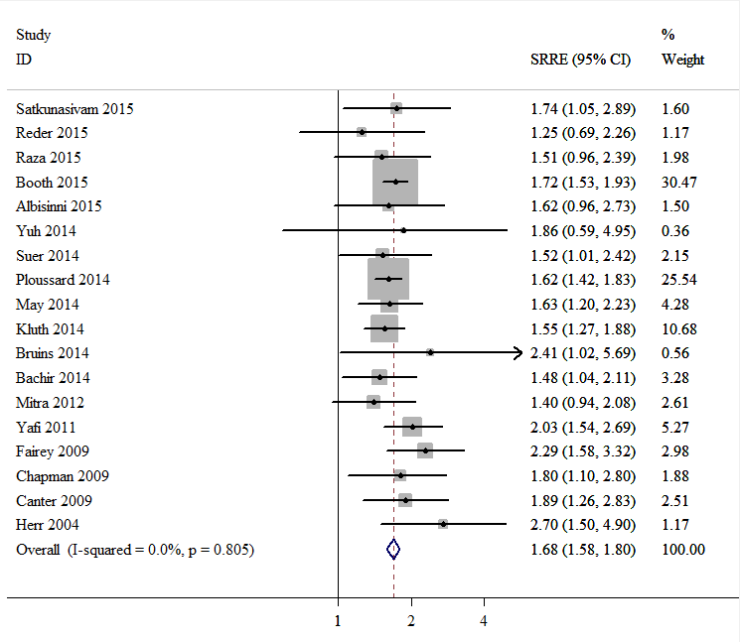
Meta-analysis of studies that examined the association between positive surgical margin and overall survival (OS) following radical cystectomy (RC)

In 16 studies, with a total sample size of 10,738 individuals, the associations between positive surgical margin and RFS of bladder cancer patients after RC were reported. A fixed effects model was used, revealing a summary relative risk estimate (SRRE) of 1.63 [95% confidence interval (CI), 1.46-1.83; Figure 2], with no significant heterogeneity found (Q statistic, *P* = 0.105; I^2^ = 32.1%). The pooled result indicated that the presence of positive surgical margins was associated with poor RFS. The CSS was reported in 26 studies that enrolled a total of 25,804 bladder cancer patients. A random effects model was used due to evidence of heterogeneity among the studies (Q statistic, *P* = 0.001; I^2^ = 54.5%). A significant CSS disadvantage was detected in the positive surgical margin group compared with the negative surgical margin group (SRRE, 1.82; 95% CI, 1.63-2.04; Figure 3). In addition, patients with positive surgical margins were found to have an increased risk in terms of OS (SRRE, 1.68; 95% CI, 1.58-1.80; Figure 4), without evidence of heterogeneity (Q statistic, *P* = 0.805; I^2^ = 0.0%).

**Figure 5:**
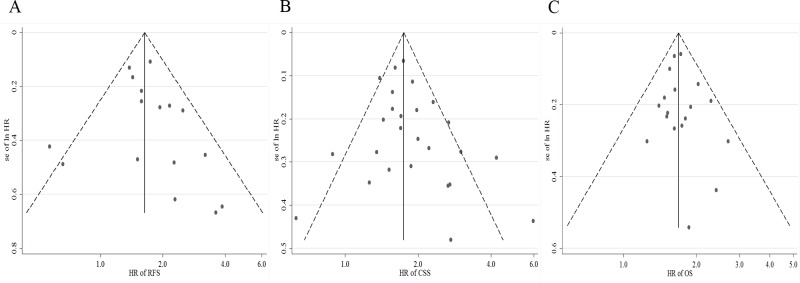
Funnel plots for publication bias of the hazard ratios (HRs) of (A) recurrence-free survival (RFS), (B) cancer-specific survival (CSS), and (C) overall survival (OS)

In sensitivity analyses excluding one study at a time, the SRRE for RFS ranged from 1.59 (95% CI, 1.38-1.83) to 1.71 (95% CI, 1.50-1.94). Similarly, the SRRE for CSS ranged from 1.77 (95% CI, 1.60-1.97) to 1.86 (95% CI, 1.65-2.09), and the SRRE for OS ranged from 1.67 (95% CI, 1.55-1.80) to 1.71 (95% CI, 1.59-1.84). These results indicated that the findings were reliable and robust. No statistical evidence of publication bias was found in this meta-analyses, as assessed by Begg’s and Egger’s tests for RFS (p-Begg = 0.300; p-Egger = 0.442; Figure 5A), CSS (p-Begg = 0.252; p-Egger = 0.194; Figure 5B) and OS (p-Begg = 0.649; p-Egger = 0.480; Figure 5C), respectively.

### Test of heterogeneity

Subgroup and meta-regression analyses were conducted to explore the source of heterogeneity according to mean patient age (≥65 *vs*. < 65), sample size (≥500 *vs*. < 500), publication year (≥2014 *vs*. < 2014), duration of follow-up (≥60 months *vs*. < 60 months), study type (single center *vs*. multicenter) and geographic region (North America, Europe or Asia). Although no significant modifiers accounting for the inter-study heterogeneity were detected, the observed heterogeneity in CSS decreased significantly in some models, such as articles published since 2014, multicenter studies, study with sample size >500 cases, and with follow-up duration >60 months. Furthermore, the results in subgroup analyses were consistent with the primary findings (Table 2).

**Table 2:** Summary of meta-analysis results for surgical margin status and outcomes of RC

Analysis specification	Studies	SRRE (95% CI)	Meta regression *P*-value	Heterogeneity I^2^	*P*-value
Recurrence-free survival					
All	16	1.63 (1.46-1.83)		32.1	0.105
Mean age					
≥65	13	1.61 (1.43-1.81)	0.259	34.8	0.104
<65	3	2.44 (1.37-4.34)		0.0	0.419
Sample size					
≥500	8	1.65 (1.45-1.87)	0.765	0.0	0.477
<500	8	1.56 (1.16-2.10)		54.6	0.031
Published year					
≥2014	12	1.73 (1.50-1.99)	0.257	3.4	0.411
<2014	4	1.45 (1.18-1.78)		65.6	0.033
Mean follow-up					
≥60	6	2.26 (1.57-3.23)	0.128	0.0	0.857
<60	10	1.57 (1.39-1.78)		46.2	0.053
Study type					
Single-center	9	1.54 (1.21-1.97)	0.624	43.8	0.076
Multicenter	7	1.66 (1.46-1.83)		20.9	0.270
Region					
America	6	1.36 (1.07-1.73)	0.160 0.306	51.0	0.070
Europe	4	1.73 (1.29-2.34)		0.0	0.861
Asia	1	3.20 (1.31-7.82)		/	/
Cancer-specific survival					
All	26	1.82 (1.63-2.04)		54.5	0.001
Mean age					
≥65	20	1.79 (1.58-2.02)	0.572	58.9	0.000
<65	6	2.00 (1.49-2.68)		36.8	0.161
Sample size					
≥500	12	1.74 (1.59-1.91)	0.662	24.8	0.200
<500	14	1.94 (1.48-2.54)		67.1	0.000
Published year					
≥2014	13	1.70 (1.53-1.89)	0.462	27.1	0.171
<2014	13	1.92 (1.54-2.40)		66.2	0.000
Mean follow-up					
≥60	4	2.30 (1.72-3.09)	0.475	0.0	0.424
<60	20	1.77 (1.54-2.04)		60.3	0.000
Study type					
Single-center	16	2.06 (1.71-2.48)	0.101	58.4	0.002
Multicenter	10	1.63 (1.45-1.84)		34.8	0.130
Region					
America	8	1.84 (1.55-2.19)	0.408 0.618	50.0	0.051
Europe	7	1.97 (1.40-2.75)		68.8	0.004
Asia	6	2.29 (1.46-3.58)		57.1	0.040
Over survival					
All	18	1.68 (1.58-1.80)		0.0	0.805
Mean age					
≥65	16	1.68 (1.58-1.80)	0.911	0.0	0.895
<65	2	1.72 (1.23-2.39)		69.3	0.071
Sample size					
≥500	9	1.68 (1.57-1.81)	0.995	0.0	0.610
<500	9	1.69 (1.44-1.97)		0.0	0.693
Published year					
≥2014	12	1.64 (1.53-1.76)	0.066	0.0	0.987
<2014	6	1.95 (1.66-2.28)		0.0	0.444
Mean follow-up					
≥60	4	1.88 (1.42-2.49)	0.980	0.0	0.434
<60	13	1.65 (1.53-1.79)		0.0	0.766
Study type					
Single-center	10	1.73 (1.57-1.90)	0.474	0.0	0.781
Multicenter	8	1.65 (1.51-1.80)		0.0	0.563
Region					
America	11	1.76 (1.61-1.92)	0.531 0.698	0.0	0.556
Europe	3	1.68 (1.31-2.17)		0.0	0.694
Asia	1	1.52 (1.01-2.42)		/	/

## DISCUSSION

RC with urinary diversion is the gold standard treatment for muscle-invasive bladder cancer or high-risk and recurrent superficial bladder cancer. According to a multi-institutional database of 888 bladder cancer patients who underwent RC, the 5-year RFS and CSS rates were 58 and 66%, respectively [42]. However, regarding bladder cancer patients with advanced tumor stage, lymph node involvement, lymphovascular invasion and high tumor grade, >50% experience systemic relapse and ∼50% develop distant metastases [43, 44]. Therefore, identifying further potential predictive markers will be useful for the prognosis and management of bladder cancer patients treated with RC.

To the best of our knowledge, the present study is the first meta-analysis of the association between positive surgical margins and outcomes of bladder cancer treated with RC. Novara G et al [6] had evaluated the prognostic relevance of surgical margin status in a multicentre study of more than 4,400 patients treated with RC. they recommended that surgical margin status should be reported in pathological reports following RC, and should prompt consideration of further adjuvant therapy for the patient. The past few years have seen growing much debate on surgical margin status and outcomes of bladder cancer after RC. However, until a prospective, randomized-controlled study is done, the findings from a meta-analysis of retrospective studies are the best evidence available.

In this analysis, 36 cohort studies were included, with a large sample size of 38,384 bladder cancer patients. This study provided relatively robust evidence demonstrating that the presence of positive surgical margins was associated with poor outcomes in terms of RFS, CSS and OS in bladder cancer patients treated with RC. The SRREs of positive surgical margins, and RFS, CSS and OS were 1.63 (95% CI, 1.46-1.83), 1.82 (95% CI, 1.63-2.04) and 1.68 (95% CI, 1.58-1.80), respectively, compared with negative surgical margins. The findings were consistently independent of age, sample size, publication year, follow-up duration, study type and geographical region. The sensitivity analyses indicated that the findings were reliable and robust. In addition, there was no evidence of significant publication bias in these analyses according to Begg’s or Egger’s tests.

Heterogeneity is often a major concern in meta-analyses. Significant heterogeneity was detected in the analysis of the CSS model, although there was no evidence of heterogeneity in terms of RFS or OS. Many confounding variables differed across the individual studies, including age, gender, pathological stage and lymph node status; this may explain the observed heterogeneity between the studies. To address the issue of heterogeneity, subgroup analyses were conducted, revealing that the significant variations were reduced in the meta-analysis of article subgroups published since 2014, with a multicenter design, with a sample size >500 patients and with follow-up duration >60 months.

Potential risk factors contributing to positive surgical margins are as follows: i) features of advanced cancer, such as lymphovascular invasion, extravesical disease and mixed histology [4]; ii) surgeon-dependent factors, including the type of procedure, technique and experience; and iii) specimen handling and accurate interpretation of RC [45, 46]. Due to the significant adverse associations between positive surgical margins and outcomes of bladder cancer after RC, interest in the preventive management of positive surgical margins has arisen. Although intraoperative frozen section analysis of the urethral margin prior to urinary tract reconstruction has been accepted as a standard practice [47], there is much debate concerning its usefulness in determining ureteral and urethral margin status [8, 48]. Further research is required to accurately evaluate the costs and benefits of intraoperative frozen section analysis for patients treated by RC.

Several important strengths of the present study should be noted. Firstly, the meta-analysis included 36 studies with a large sample size to detect more stable associations and provide more reliable results. Secondly, strict accordance with the inclusion and exclusion criteria was maintained, and we also extracted available data from relevant studies that mentioned the relationship between surgical margin status and survival outcomes of bladder cancer patients after RC. Furthermore, the results were found to be reliable and robust through subgroup and sensitivity analyses.

However, the study was subject to several limitations. First and foremost, the majority of the included studies were retrospective cohort studies, which made our meta-analysis sensitive to potential confounding variables. Additionally, although the results from the main multivariable model that included the most adjusted confounders were used, there may be residual or unknown confounding variables that were not taken into consideration in the included studies. Secondly, substantial heterogeneity was observed in the meta-analysis of CSS. Although subgroup and meta-regression analyses were conducted to explore the source of heterogeneity, no effect modifier of heterogeneity was found. Thirdly, we were unable to explore the potential differences in associations according to the classification of bladder cancer. It remains unknown whether the findings may vary by tumor subtype or tumor stage, even though some subgroup analyses were conducted. An additional limitation is that the detection methods of surgical margin status were not definitely described in the majority of the included studies. Therefore, a subgroup analysis for detection methods could not be performed. Moreover, other detailed information regarding the features of the margins, such as the location, focality, and microscopic or macroscopic features, were also not presented in the included studies, and could not be further examined.

In summary, the present meta-analysis confirms that bladder cancer patients with positive surgical margins, as compared with negative surgical margins, are likely to have poorer RFS, CSS and OS after RC, indicating that surgical margin status may be an independent indicator of the survival outcome of patients with bladder cancer following RC.

## MATERIALS AND METHODS

### Search strategy

This meta-analysis was performed in accordance with the Preferred Reporting Items for Systematic Reviews and Meta-analyses (PRISMA) guidelines [49]. A systematic literature search was performed in the Pubmed, Embase and the Cochrane Library databases to identify eligible studies published between the inception of the databases and April 2016. The primary search string included the following items: ‘bladder cancer’, ‘transitional cell carcinoma’ or ‘urinary bladder neoplasms’; ‘margin’ or ‘margins’; ‘surgery’ or ‘radical cystectomy’. The search was focused on human studies. No additional filters were included to restrict the search. Furthermore, a manual search of the reference lists of relevant review articles was conducted to identify all available studies.

### Inclusion and exclusion criteria

The eligibility of each study was assessed using the population, intervention, comparator, outcome and study design (PICOS) approach [49]. A study was included in the analysis if it met the following criteria: the bladder cancer patient was treated with RC (P); the surgical margin was assessed by pathologists (I); the oncological outcomes with positive surgical margins were compared with negative surgical margins (C); the results were reported as risk estimates (hazard ratios, risk ratios, odds ratios) with corresponding 95% confidence intervals, or sufficient data was provided to estimate these (O); and the study was a prospective or retrospective cohort design (S).

In addition to these criteria, only studies that reported survival outcomes, such as RFS, CSS or OS, were considered for inclusion. Case-reports, reviews, expert opinions or meeting abstracts without usable data were excluded. In studies with the same or overlapping population, only the most recent and informative study was included in the meta-analysis.

### Data extraction

Data from all included studies were independently extracted by two investigators and corroborated by another investigator. The following information was extracted for each included study: first author, year of publication, study type, country, study period, duration of follow-up, sample size, mean age, gender, pathological stage, positive lymph node rate, positive surgical margin rate, and risk estimates of RFS, CSS or OS based on margin status. If one study contained multiple data sets, the one with more adjusted confounders was used. All discrepancies in data extraction were resolved by discussion.

### Statistical analysis

All included studies used multivariable Cox proportional hazards ratio models to examine the associations between surgical margin status and survival outcomes of RC, including RFS, CSS or OS. SRREs and 95% CIs were calculated in order to compare the positive margin group with the negative margin group. Heterogeneity between studies was assessed using the Q and I^2^ statistics. *P* < 0.10 or I^2^>50% were used to indicate heterogeneity. Random effects models were used for meta-analysis in cases of heterogeneity. Forest plots were also applied to assess the relationships between margin status and survival outcomes of bladder cancer treated by RC.

Subgroup and meta-regression analyses were performed to examine potential sources of heterogeneity according to age, sample size, publication year, follow-up duration, study type and geographical region. Sensitivity analyses were conducted to assess the robustness of the results by repeating the meta-analysis after omitting one study at a time. Furthermore, funnel plots were inspected for asymmetry, and Begg’s and Egger’s tests were used to assess publication bias. All statistical analyses were conducted using STATA 12.0 software (StataCorp LP, College Station, TX, USA). *P* < 0.05 was considered to be an indicator of significance, except where specifically noted.
